# Same Journey, Different Paths: Caregiver Burden among Informal Caregivers of Adolescent and Young Adult Patients with an Uncertain or Poor Cancer Prognosis (UPCP)

**DOI:** 10.3390/jcm13010158

**Published:** 2023-12-27

**Authors:** Milou J. P. Reuvers, Vivian W. G. Burgers, Carla Vlooswijk, Bram Verhees, Olga Husson, Winette T. A. van der Graaf

**Affiliations:** 1 Division of Psychosocial Research and Epidemiology, Netherlands Cancer Institute, 1006 BE Amsterdam, The Netherlands; v.burgers@nki.nl (V.W.G.B.); o.husson@nki.nl (O.H.); 2 Department of Medical Oncology, Netherlands Cancer Institute—Antoni van Leeuwenhoek, 1006 BE Amsterdam, The Netherlands; w.vd.graaf@nki.nl; 3 Department of Medical Oncology, Erasmus MC Cancer Institute, Erasmus University Medical Center, 3015 GD Rotterdam, The Netherlands; 4 Department of Research and Development, Netherlands Comprehensive Cancer Organization, 3511 DT Utrecht, The Netherlands; 5 Hoestie Foundation, 5616 JX Eindhoven, The Netherlands; 6 Department of Surgical Oncology, Erasmus MC Cancer Institute, Erasmus University Medical Center, 3015 GD Rotterdam, The Netherlands

**Keywords:** informal caregivers, adolescent and young adult oncology, caregiver burden, advanced cancer

## Abstract

A minority of adolescent and young adult cancer patients (AYA) live with an uncertain or poor prognosis (UPCP). Caring for a young, advanced cancer patient can lead to caregiver burden. This study aims to provide insight into burden on informal caregivers of AYA cancer patients with UPCP. In-depth, semistructured interviews were conducted with parents (*n* = 12), siblings (*n* = 7), friends (*n* = 7), and partners (*n* = 13). Thematic analysis was performed to derive themes from the data. Participants reported sleeping problems and stress. They struggle with uncertainty, fear, loss, and negative emotions. Family life is altered due to solely taking care of the children, but also the AYA. Contact with friends and family is changed. The relationship to the AYA can shift positively (e.g., becoming closer) or negatively (e.g., more conflict or no longer being attracted). Participants were under pressure, having to take on many responsibilities and multiple roles. In the financial domain, they report less income and often must continue working. A high amount of caregiver burden is experienced among informal caregivers of AYAs with UPCP. Yet only part of the impact appears to be age specific. Specific, age-adjusted interventions can be developed to lower the burden.

## 1. Introduction

Adolescents and young adults (AYA) with cancer are patients diagnosed between the ages of 15 and 39 years [1]. These patients encounter various psychological challenges, as they are diagnosed during a developmental period in their lives during which many alterations occur (for example, starting a family and a career) [2]. The vast majority are diagnosed during a stage at which treatment with curative intent is still possible, albeit often requiring intensive treatments [3]. However, a subgroup of AYA cancer patients lives with an uncertain or poor cancer prognosis (UPCP). Burgers and colleagues defined them as “patients with advanced cancer for which there is no reasonable hope of cure, indicating that they will die prematurely from cancer, but have no immediate threat of death” [3]. These patients can be divided into three groups: patients on newer therapies (immunotherapy, targeted therapy), those on traditional therapies (chemotherapy, radiotherapy), or patients diagnosed with a low-grade glioma (on a wait-and-scan policy after surgery). Due to their uncertain life expectancy, questions regarding the future, remaining treatment options, and the essence of their lives emerge in AYA cancer patients with a UPCP [3].

AYA cancer patients oftentimes become dependent on their informal caregivers, as they frequently face a reduction in autonomy due to disease symptoms and side effects of their treatment [3]. Consequently, taking care of an AYA cancer patient with a UPCP could result in caregiver burden, defined as “the extent to which caregivers perceive that their physical health, psychological health, schedule, social life, and financial status have suffered due to providing care for a cancer patient” [4]. Our recent review shows that informal caregivers of AYA cancer patients experience impact in each of the domains of caregiver burden [5]. Aside from the fact that caring for a patient of AYA-age is already demanding, research shows that taking care of someone with advanced disease could entail an additional burden. A study among informal caregivers of advanced cancer patients stated that having to observe someone’s physical deterioration was difficult and caused them to feel helpless. Uncertainty about the future was perceived as straining among these caregivers. They also reported that providing emotional support was considered more demanding than providing practical help [6].

Informal caregivers of AYA cancer patients are involved in many day-to-day tasks and adopt multiple roles. They provide practical as well as emotional support for the patient. Caregivers oftentimes report negative emotional states related to caretaking and they face age-specific challenges related to the young age of the patient (e.g., an altered future for their partners) [5]. Consequently, the diagnosis and disease trajectory may affect the relationship with their caregivers. Physical and/or emotional intimacy may decrease, or conflicts can arise. These changes in the relationship are often related to the patient’s physical decline and the growing pressure on the caregiver. If communication is perceived as challenging, these negative feelings can intensify, and divergence may occur [7]. In addition, AYA cancer patients with a UPCP report that they are not undergoing similar development to their peers. Moreover, these peers frequently cannot relate to the patient’s circumstances and therefore cannot provide sufficient support [3]. This leads to a high risk for social isolation, which can make a patient more prone to negative physiological responses, such as hypertension [8]. 

Informal caregivers are an understudied group in AYA literature as the focus is typically on the needs and associated problems of the patients. In addition, caregivers taking care of advanced cancer patients are different from those dealing with curative intent. There is no literature on the wellbeing of caregivers for AYA cancer patients with a UPCP, although they face age- and disease-specific burden as caregivers while coping with the uncertain life expectancy. Creating an understanding of the challenges this group experiences in daily life can help to adequately support them by providing or developing interventions, and information to cope. This might increase their quality of life, both before and, when applicable, after the death of AYA cancer patient. In addition, the wellbeing of the informal caregiver seems to be related to the patients’ health. Since current care initiatives are not tailored to this group of caregivers, it is important to align their impact with the content of these programs. Therefore, this interview study aims to identify the impact on daily life of a caregiver of an AYA cancer patient with a UPCP, using the definition of caregiver burden to determine the impact.

## 2. Materials and Methods

The Consolidation Criteria for Reporting Qualitative Studies guideline was followed to guarantee quality and transparency of reporting [9].

### 2.1. Sample and Procedure

A qualitative, descriptive study was performed to examine the impact of cancer and the prognosis on caregivers of AYA cancer patients with a UPCP. Informal caregivers were defined as “the individuals that you would turn to for help in making decisions and depend on to be with you when getting your cancer care”. Caregivers were invited to participate by an AYA who participated in the INVAYA-study or via word-of-mouth. Patients were asked during their interview to invite one or more of their informal caregivers to participate and had 1–2 weeks to nominate them. The patient received information via e-mail regarding the caregiver part of the study. During the patients’ follow-up call, they were asked for consent to contact their informal caregiver(s) and to receive the caregivers’ contact information. AYAs were also able to indicate their preference on how to approach the caregivers (via telephone or e-mail). Caregivers were contacted by the researcher, provided with information, consent was gathered, and a date for the interview was set. Caregivers had to be able to speak and understand the Dutch language. Semistructured interviews were conducted among these informal caregivers of the AYA cancer patients, stratified into four groups (parents, partners, friends, and siblings) until an equal distribution of the type of caregivers was reached. An effort was made to interview as many different types of caregivers as possible. Since partners and parents are the ones exhibiting the most prominent role in the care of these patients, they were represented more frequently in the sample.

Participants were asked open-ended questions regarding the impact on their daily lives, their relationship to the patient, and their support preferences (Table A1). The interview guide was derived from the interviews conducted with AYA cancer patients and adapted to specify the impact on caregivers. Five AYA research partners were subsequently asked what they would like to know from their informal caregivers. This input was transformed into questions and added to the guide. Prior to the interview, the caregiver completed a case report form (CRF), answering questions on their age, living situation, employment status, educational level, and work status. In total, 36 interviews were conducted, which ranged between 36 and 112 min (mean duration: 59 min). All interviews took place via Microsoft Teams due to COVID-19 restrictions. One to two weeks after the interview, caregivers had a follow-up call to evaluate the interview and were allowed to share information they had forgotten. These interviews were part of the INVAYA-study, which was approved by the Institutional Review Board of the Antoni van Leeuwenhoek hospital in Amsterdam, the Netherlands (IRBd20-205). The INVAYA-study focused on AYA cancer patients coping with a UPCP, their caregivers, and health-care professionals [10].

### 2.2. Data Analysis

In-depth interviews were conducted by a female psychologist and researcher (V.B.), a female researcher (C.V.), and a female research assistant (E.D.), and were audiotaped and transcribed verbatim. A second researcher (M.R.) analyzed the interviews using QSR NVIVO [11]. The analysis was based on the bottom-up thematic analysis from Braun and Clarke [12]. First, all interviews were reviewed twice, after which all textual information describing any type of impact was highlighted (open coding). The codes gathered from the data were divided among the different categories of caregiver burden: biological, psychological, social, schedule, or financial (Table A2). Four independent researchers then examined which of the codes were considered AYA-specific and which applied to caregivers of an AYA cancer patient with a UPCP in particular until they reached consensus. A subsequent analysis of these codes was conducted to discover overarching themes (axial coding). Descriptive statistics were used to determine the sociodemographic data, which was performed using SPSS version 26.0.

## 3. Results

Forty-two caregivers were nominated by AYA cancer patients. In total, 39 caregivers were interviewed. The remaining three caregivers eventually opted out before their interview. Three interviews were conducted with both parents attending, resulting in thirty-six interviews in total. These included 13 partners, 12 parents, 7 friends, and 7 siblings. Table 1 presents the characteristics of the participants. Quotes supporting the data are presented in Table A3.

### 3.1. Biological Impact

Caregivers reported that they experienced **reduced energy**. This was mainly due to sleeping problems, partly due to worrying about the patient or tasks that must be accomplished (e.g., maintaining the medication schedule). Some caregivers also mentioned that they were on sick-leave and had difficulty concentrating. **Stress** had a significant impact and led to being less able to handle crowds and occasional panic attacks. Stress also led to physical symptoms. For example, a sister reported having to go to her general practitioner for chest pain, and a partners’ psoriasis exacerbated. Also, **changes in lifestyle** tended to occur. A mother mentioned that she had reduced time and ability to properly care for herself and to be physically active. Aside from that, caregivers reported that it was more difficult to be active, as the patient was not able to perform many physical activities. One caregiver reported that she was **not attracted** to her partner anymore. This was partly because he had changed from a young, active person to a frail man with an altered appearance. This partner did not mention this to the patient, in order not to hurt him. This also impacted their sexual relationship, as the caregiver reported a decreased interest in sex (1.1). This issue is multifaceted, as it is both psychological and social in nature.

### 3.2. Psychological Impact

The informal caregivers reported **fear** of the patient’s declining health status, the end-of-life phase, death and being left alone. It was **confronting** for them to be engaged in conversations regarding death and to prepare for it (e.g., writing the will). Also, being in support groups with others in a similar situation was burdensome for some of them. For parents, having to take care of their sick adult children was confronting and felt unfair (2.1). **Uncertainty** related to prognosis, upcoming disease progression and life expectancy oftentimes occurred. Furthermore, having to wait a long time for scans and the results, and not knowing how to best fulfill their lives (2.2) had a big impact on caregivers. Due to the patient’s illness, caregivers became more aware of their own mortality. 

**Negative emotions were** evoked because of caregiving. Informal caregivers reported guilt toward the patient because the disease had such an impact on their lives and had difficulty balancing and respecting the patients’ independence when taking care of them. They worried greatly about the patient, but also tried not to burden them. Caregivers of patients with brain tumors coped with the frustration of navigating the patient’s cognitive impairment. Patients also tended to share less information than caregivers preferred, which also led to frustration. Caregivers reported their lives being put on hold and fully devoting themselves to the patient, while ignoring their own needs (2.3). Parents and a sister mentioned not being able to enjoy their lives as much as they had before the cancer diagnosis. Furthermore, many caregivers felt that they were not able to accept the disease and its prognosis. Caregivers reported high levels of **hopelessness**, as they were unable to support the patient’s need to manage their psychological symptoms, to prevent or mitigate physical decline, and felt unable to adequately provide help during an emergency, in juxtaposition to their commitment to provide the best care possible. Caregivers were also burdened by the idea of the patient being unable to go through all the phases of life.

Caregivers reported an **impact on their family life**. They identified difficulty with being left alone with children when the partner dies. Caregivers struggled with the fact that their children would be growing up with only one parent, or that the child would be too young to remember the patient as their parent later in life. Partners were also afraid of falling ill themselves and leaving their children orphaned. Caregivers were burdened by the knowledge that their **young children** were aware of and sad about the disease, and they feared the children would not be able to deal with the death of their parent (2.4). Moreover, caregivers were occupied with not burdening their children with the disease and its impact. Furthermore, a major impact was seen on the **ability to start or enlarge one’s family**. For many partners, it was difficult that the possibility of having children had been taken away, but also that no fertility preservation had occurred prior to treatment (2.5). It was also challenging for them to talk about fertility at such a young age. Friends and siblings reported feeling that the patient was not able to enjoy being with their children, or that they were not able to tell the AYA that they wanted to start a family because it felt inappropriate and burdensome.

**Loss** was described in various ways. Caregivers mentioned that they felt too young to be in this situation and that their future would change due to the disease. Siblings perceived difficulty with being left as the only child once the patient has died. Partners worried they would never find a new partner that would meet their needs and/or accept their situation. Furthermore, caregivers found it challenging that they were not to be able to do as much as their peers. It was burdensome to them when the patient was not able to accept their disease. 

### 3.3. Social Impact

Caregivers reported **difficulties in contacting others** (3.1). It was difficult for them to realize they often cannot relate to others in support groups, as some of these patients and their caregivers were already going into the end-of-life phase. Moreover, caregivers tended not to talk to others about the disease, to avoid being a burden to them. They found it difficult to explain to their young children what is happening and what would happen in the future. Often, partners and AYAs had to discuss together what they wanted to share with their children or did not talk about the disease in their children’s presence. Sometimes caregivers felt like they only received pity in their contact with others, even though they wanted to be approached normally. Sometimes they isolated from others to reduce the risk in infecting the patient, as this study was conducted during the COVID-19 pandemic. Subsequently, others often also did not want to talk about the disease (for example, with their children), were happier with the scan results than patient and caregivers, or did not talk about their own lives to unburden them. Furthermore, caregivers experienced unsolicited relationship advice from those who tended to interfere, as they did not want the caregiver to experience the pain of losing someone. 

**Friends** were an important source of support. However, it was more difficult for caregivers to meet their friends regularly, as they had less leisure time. Caregivers sometimes quit their hobbies to fully devote themselves to caregiving, resulting in less time for themselves. In addition, they experienced that their friends had difficulty **relating and empathizing to the situation**, oftentimes due to their relatively young age and lack of familiarity with similar situations. They could not compare themselves to other AYAs on curative treatment and their caregivers or to older patients with a similar prognosis (3.2). Caregivers reported that support decreased through time since the diagnosis was longer (3.3).

Caregivers had to make many **adjustments in their relationship to the AYA**. For instance, they had to adapt to when the patient wanted to talk about the disease and felt burdened in addressing it. They also did not want to strain the patient with discussions about difficult topics. It was challenging for them to empathize with the AYA’s situation, and they perceived that they were experiencing a different trajectory (3.4). The disparate impact on their own life also made caregivers want to hear different information regarding the prognosis. They were less likely to cancel the plans with the AYA due to the limited time they have left together. Caregivers mentioned that asking how they and the patient are doing was often seen as **adequate support**: e.g., “just reach out”. They mentioned that being able to talk about what they are dealing with, as well as doing fun activities with friends as a distraction, was helpful. They also obtained support from others who were in a similar situation. Caregivers working in a medical environment mentioned that sharing their experiences with individuals with medical knowledge was often easier for them to share their experiences. 

### 3.4. Schedule Impact

Caregivers performed **various caregiving tasks**, which they undertook in addition to their own daily life activities (e.g., employment, their family, household). They often accompanied the patient to appointments, both as transportation and to support them for the conversation with the treating physician. Prior to the consult, they helped to structure what questions they had, and made sure these were answered. Caregivers often completely took over household tasks, as the patient often had little energy or wanted to spend their time and energy differently. Caregivers planned out the days and made sure the patient maintained their medication schedule. In addition, they tried to distract the patient by bringing them along and engaging in various activities. 

**Difficulties in taking on multiple roles** were also reported. Being both a partner and a caregiver was considered challenging. Additionally, the combination of being employed and taking on caregiving tasks appeared to be a significant burden. The impact on the caregivers’ **family life** sometimes changed due to the disease. Partners of AYA cancer patients often were their children’s sole caregiver. In some cases, both the patient and children must be cared for by the informal caregiver. Others (e.g., parents or siblings) frequently took on a larger role in the care of the patient’s children. Consequently, their own daily life or work situation had to be adjusted. 

There were **sacrifices** to support the AYA cancer patient. Their own plans were often cancelled in order to stay with or take care of the patient, and making plans for the future was no longer possible (4.1) due to the uncertainties associated with the disease and the patient’s unpredictable physical condition. Traveling, in general, was cancelled to stay close in case the AYA needed medical assistance. An AYA and partner decided not to emigrate because of cancer. This also complicated engaging in spontaneous activities. Sometimes caregivers had to (temporarily) stop working or move in with the patient to assist with caretaking. Their personal time was sacrificed as a result (4.2). 

**Other changes related to the future** involved earlier marriage, so that decision-making would become easier (4.3). Some female caregivers indicated they wanted to become pregnant more quickly to be more certain that the patient would still be alive long enough to meet the child. One partner indicated they did not start new education because it was not possible to combine it with caregiving. Study delay also occurred because caregiving led to reduced time to focus on their studies. Some caregivers reported bringing their wedding forward so that the AYA could be present and they could celebrate this milestone together. In addition, caregivers with medical knowledge stepped in to navigate healthcare and conversations with the medical team. Caregivers tried to prepare for their changed future, as after death the patient’s would be the sole caretaker of their children (4.4).

### 3.5. Financial Impact

Caregivers worried about **reduced income**, as most patients were no longer able to stay employed and their benefit oftentimes did not cover the loss of their salary. Managing on one income was perceived as challenging by caregivers (5.1) and resulted in anxiety and stress. Due to the decrease in combined income, for some there was little room to engage in recreational activities. Caregivers occasionally needed to find a different job to guarantee enough income and cover the loss of financial resources. Applying for benefits turned out to be difficult and burdensome. Caregivers would prefer to spend less time in employment and more time on activities they enjoy doing or staying with the patient in their remaining time (5.2) but were often not able to arrange this financially.

As both the patient and caregiver, due to their young age, have often not accumulated as many resources in terms of financial security, the financial impact was great and motivated **decision-making**. In some cases, this led to the decision to marry to make financial decisions easier. They also experienced that obtaining a mortgage was more difficult or impossible, which in some cases meant that people had to rent and were not able to buy a house (5.3). In some cases, caregivers had to sell their house as the mortgage had become too expensive given their reduced income. 

## 4. Discussion

This qualitative, explorative study aimed to identify age-specific impact on caregivers of AYA cancer patients managing a UPCP. The impact on these caregivers was present in all domains of caregiver burden: biological, psychological, social, scheduling, and financial. Some of the burdens might be interpreted as age-specific, such as the inability to start or extend a family and the anticipated grief in having to care for the family alone after death. Also, the lack of understanding from peers and the (permanent) change in the future of caregivers are also more profound among the interviewees, which may have a different impact in comparison to those taking care of pediatric or older patients. In addition, the young patients and their partners might have comparatively few financial resources and, consequently, the impact on their assets can be high. However, most of the challenges described by the informal caregivers were not interpreted to be AYA-specific, nor specifically related to cancer. The firm impression of the authors is that the impact on psychological and schedule domain was greater than in the other domains and age-ranges. However, this is not quantitatively examined.

In line with our results, Junkins and colleagues report that caregivers of AYA cancer patients do not only exclusively experience challenges that are related to the specific age of these relatively young patients [13]. Caregivers of AYA cancer patients also dealt with some of the same problems as other caregivers do (for example, changes in intimacy, which also occurs in older adult dyads) and, in addition, the caregivers report universal experiences (e.g., impact on financial situation, changes in relationships to others, and need for additional support) [13]. Caregiver burden is also dependent on patient and caregiver related factors. The literature shows that factors that are associated with increased caregiver burden include lower educational level of both caregiver and patient, employment and income, low social support, comorbidities of the patient [14], treatment period and type, psychological symptoms [15], caregiver age [16], gender, and the amount of time spent caregiving [17]. Also, nonwhite caregivers tend to report more caregiver burden [18]. Due to the qualitative nature of this study, there was no opportunity to examine this aspect.

Several caregivers reported the need for a preplanned psychosocial track, as the psychological burden is substantial. Here, focus can be placed on fears and negative feelings that arise from caregiving for a young patient with a poor cancer prognosis. These negative feelings are also mentioned among other informal caregivers of AYAs [19,20,21,22]. Psychological consultations (one-on-one) during palliative trajectories can reduce stress and improve coping and communication with the patient [23,24]. AYAs and caregivers in this study indicated that talking to one another about the situation is perceived as therapeutic. This opens a mandatory conversation and addresses topics that may not usually be appointed, as this is difficult for informal caregivers to initiate [25,26]. An intervention opportunity would therefore be to have a session (at least once) with their healthcare professional (HCP), partner, and AYA to discuss age-specific topics or receive more information, aside from the normative consultations. Regarding schedule impact, it is difficult to create room and opportunities for caregiving without having to combine it with multiple other roles in daily life. However, caregiver training and skill development interventions might assist caregivers in growing into the caregiver role. Psychoeducational interventions can also help caregivers to adjust to their role and feel confident and self-efficacy when performing tasks [27,28]. 

Current AYA-care in the Netherlands could be complemented with care for these caregivers, considering that this study clearly shows that the burden on this group is profound, with negative impact on the patient’s and caregiver’s wellbeing. Given that challenges are not always age specific, aspects and interventions that decrease caregiver burden in the general population could also be implemented within caregivers of AYA with a UPCP. Social support can be an important component, together with teaching adaptive coping strategies. In the literature, this had a positive effect on the caregivers’ emotional and physical wellbeing [29]. The use of social media can help them to find similar experiences and seek support from others in a similar situation and is used often in this young population. Digital interventions can help to achieve adequate communication with each other and improve decision-making [30]. However, for some of the caregiver burden described in this article, there does not yet seem to be a ready-made solution. Experiencing a changed future and not reaching milestones (raising a family, buying a house) cannot be solved and will continue to be a frequently reported matter of fact among (mainly) partners.

This paper is the first to present how caregivers of AYA cancer patients managing a UPCP experience the impact of the disease on their daily life, and describes the perceived caregiver burden among this group. Moreover, for a qualitative study, the sample size was relatively large, resulting in different types of caregivers providing in-depth information on the impact on their life. Aside from the strengths of this study, there are also limitations. Social desirability could have occurred because caregivers are concerned that patients would be able to read the results of the study. Qualitative research is susceptible to interpretation by researchers, indicating that certain outcomes may have been interpreted differently than the interviewee intended. Also, due to the qualitative nature of the study, it is more difficult to generalize the results, and no factors associated with caregiver burden could be determined. Hence, what the emphasis should be when providing support cannot be properly examined. The results of this study cannot be generalized, as limited caregivers with a migration background participated and the educational level of the participants was high. Also, the sample that was interviewed shapes the results and therefore may provide a bias. Depending on their problem, results are presented. A different sample may lead to different results, for example, if their patient is doing worse or when informal caregivers coping with other tumor types are included.

## 5. Conclusions

Caregivers of AYA cancer patients with a UPCP experience burden in multiple domains of daily life. The burden is extensive and the issues mentioned can be included in follow-up research, identifying which issues require specific interventions. HCP can be encouraged to properly refer these caregivers for appropriate psychological support or to organizations to give practical support. It may lead to fruitful conversations, encourage their informal caregivers to seek additional help or motivate the AYA patient to seek further support to unburden caregivers. By supporting caregivers, their burden can be reduced and their quality of life improved. This can enable caregivers to provide better care of the patient, which also might enhance the patient’s quality of life.

## Figures and Tables

**Table 1:** Sociodemographic information of caregivers of AYA cancer patients with a UPCP.

	N	%
Mean age (±SD) 41.8 (13.77)		
Sex		
Female	26	66.7
Male	13	33.3
Marital status		
Married/with	38	97.4
partner		
Single	1	2.6
Living situation *		
With partner	35	89.7
With children < 18	18	46.2
With children > 18	1	2.6
Alone	2	5.1
Other	1	2.6
Educational level		
Secondary education or less	1	2.6
Secondary vocational education	13	33.3
Applied university	19	48.7
University	6	15.4
Employment status *		
Full-time	16	41.0
Part-time	15	38.5
Unemployed	3	7.7
Sick-leave/disabled	2	5.1
Retired	4	10.3
Other	1	2.6
Relationship to the patient		
Partner	13	33.3
Parent	12	30.8
Friend	7	17.9
Sibling	7	17.9

* Participants were able to select multiple answers.

## Data Availability

Data will not be made available due to privacy reasons of the participants.

## Appendix A

**Table A1:** Interview guide.

Questions	Probes
1. How did the disease of your AYA impact your life? (e.g., daily life, major life decisions)	Education, employment, finances, hobbies, being independent, social life, family life
2. Do you think that the impact would have been different if your AYA would get better/would be curatively treated?	
3. In what way did the disease of your AYA impact your relationship? 4. Did your relationship change and, if so, how? Who among you has changed?	Sexuality, intimacy, roles/relationships, equality, tasks, financial independency, communication
5. How do you cope with the impact of the disease on your life? What would help you to deal with this more effectively? 6. How could your AYA best support you in this regard?	
7. How do you experience the support you receive? 8. How do you think you and your AYA can best support/help each other?	To what extent, in what way, frequency, and from whom do you experience support? What could they do better? Is there support you are missing/ have been missing? What advice do you have for your support system? What would be the best way to support you right now?
9. How has your perspective on the future changed as a result of the diagnosis? 10. How do you perceive the future? 11. What are your needs regarding communication about the future?	How do you see the future? What would you prefer based on the current situation? What concerns you the most? What are you afraid of? Did this change over time? With whom can you (not) communicate about this? How do you talk about it then? With whom would you like to communicate about it?

**Table A2:** All codes derived from interviews with caregivers of AYA cancer patients with UPCP.

**1.** **Biological impact**	**Mentioned by**
1.1 Energy reduction—fatigue	
Difficulty sleeping	Mothers
Performing work tasks costs more energy	Sister
Fatigue	Partners
Sleeping problems because AYA is in pain	Partner
Sleeping problems because I worry about practical things	Mothers
Worrying costs a lot of energy	Mother
1.2 Physical symptoms	
Being less productive	Sister
I cannot concentrate anymore	Mother
I cannot handle crowds	Sister
Physical symptoms as a result of stress	Sister, partner
Stress to keep the medication schedule going	Partner
1.3 Lifestyle changes	
Difficult to take care of self	Partner
I am not physically active	Mother
I do not feel attracted to my partner anymore	Partner
Living healthier because I will be living alone with the children	Partner
Not being as physically active because AYA cannot physically handle that	Partner
**2.** **Psychological impact**	
2.1 Difficulty losing AYA or being alone	
Anxiety about losing AYA	Partner
Difficulty facing that I will be left alone with the children	Partners
Difficulty facing that I will lose my only sibling and be alone	Sister
Fear of what is left of me after AYA passes	Partners
I am afraid of the moment when AYA will pass	Partner
I cannot imagine a life without AYA	Partner
I do not want to be alone	Partners, mother
I do not want to think about life after death	Mothers, sisters, father, partners, friend
I get emotional thinking about being alone	Partners, mother, sister
I worry that I will never find a partner like AYA again	Partner
I would not want to know when AYA will pass	Partner
I am trying to get used to the idea of losing AYA	Sister
If I think about life after death, it feels like I am giving up on this one	Partner
I am scared because eventually decline will occur	Partners, sister, parents
When we receive bad news, I think about death	Sister
2.2 Confrontation	
Confronting having to arrange things regarding death	Partners, mother, fathers
AYA cannot enjoy us having children	Brother
The disease is constantly on my mind	Mothers, father, partners
I cannot provide emotional support because we are too close	Father, partner
Confronting that the children are aware and sad about the disease	Partners, mother
Confronting when others deal with the same disease	Partners, mother, friend
Confronting having to take care of my sick child	Parents
Difficulty having to discuss death-related topics	Fathers, mother, partners, sisters, brother
Difficulty to realize own mortality	Friends, sister
Doing my work is very confronting	Sisters, partner
Everything we do together could be for the last time	Mothers
Going to hospital appointments is confronting	Mother, friend
Difficulty to see AYA does not have a partner because of their illness	Parents
Having to talk about fertility	Mother, partner
Having to talk about death is confronting	Partners, father, sister, mother
I can only talk about the situation on superficial level, otherwise I get too emotional	Mothers
I cannot deal with seeing my parents sad	Partner, friend
Seeing the surgical scar is very confronting	Mother
Sometimes thinking about death overwhelms me	Sisters, brother
Support groups are very confronting	Father, mothers, partners, sister
We had to discuss a different term for the disease because it was too confronting	Partner
Trying not to burden children with disease	Partners
2.3 Uncertainty	
Being emotional because of the uncertain prognosis	Partners, parents
Difficulty dealing with an uncertain prognosis but also living life to the fullest	Mother, sister
Every day I feel different	Partner
Experiencing a sense of life-urgency	Partner, sister
Fear of disease progression	Partners, parents, sister, friend
The future is based on period in between scans	Mothers, partners,
Hyperfocus on negative scan result and insecurity	Partner, friend
I do not want to think about the future because I am scared	Partners, mother
I am aware nothing is certain in life	Friend
Insecurity about what the next treatment will be	Mother
Scanxiety	Mothers, partners, sisters, father
The future is very uncertain	Partners, sisters, parents
Uncertainty about what to do with work	Partners
Uncertainty about surgery and its risks was very hard on us	Friend
Uncertainty about how long life will be	Partners, mothers, father, sisters, brother
Worried where I have to live when AYA passes	Partner
2.4 Feeling helpless	
Difficulty dealing with AYA not accepting the disease and asking questions	Mother, partner
Difficulty being unable to help AYA during their psychological process	Partner
Difficulty to see AYA struggle	Mothers, father, partners, friend
I do not want to see AYA progressively getting worse	Sister, partners
I feel helpless	Friends, partners, mothers, sister,
I feel scared because I do not know how to handle all (urgent) situations	Partner
I feel unsteady	Mother
Worried I cannot help AYA during the end-of-life phase	Partner
Worried AYA will suffer	Mother, partners
Mood swings (AYA) are directed at me	Partner, mother
2.5 Having a changed future	
Difficulty dealing with a changed future	Mother, partner, friend
Difficulty knowing you cannot have children	Partners
Difficulty confronting that we did not do fertility preservations	Partner
I do not want to spend as much time of my life working	Sisters, partners
I feel too young to be in this situation	Partner
Life is put on hold	Partner
2.6 Experiencing negative emotions	
Feeling left out because AYAs priority is their family	Parents
Disease causes us not to enjoy life anymore	Mother, sister
Feeling lonely even though others reach out	Partner
Feeling sad my children will never know AYA	Sister
I am not very open regarding my emotions	Partners
I cannot accept the situation	Parents, sister, partners
I cannot deal with negative emotions	Sister
I cannot express sadness	Partner
I cannot take as much since AYA got sick	Mother
I could not work because I cannot deal with emotional disruptions	Partner
I did not take the time to process everything	Partners
I feel like I am not myself	Sister
I wonder if I need to ask more questions and listen more	Brother
I think and worry a lot about the disease	Partners, mother, sister, friend
I wonder why this is happening to me	Sisters
I am less happy than I was before she got sick	Partner
Informal care was emotionally challenging	Friend
Feeling guilty because it has such a big impact on my life	Sister
It feels unfair that my child is sick and not me	Parents
It feels unfair that we have to deal with this	Mother, father, sisters, partner
Hurts that they are so young and has to deal with this disease and death	Mothers, father, friend
It is difficult that AYA will not move through all phases of life	Brother
No one asks me if I want to continue living	Partner
Work issues feel very irrelevant to me, and I cannot deal with them	Partners, mother, sister
I do not care as much about what others think	Mother
2.7 Worrying	
Afraid that my unborn son will inherit my sadness	Sister
Concerns because AYA is self-employed and uninsured	Mother, partner
Difficult to tell AYA we want to have children as she cannot have any	Brother, friend
Fear to get sick myself and leave children without parents	Partners, friend
I wonder if they made life choices that caused this disease	Sister
I worry if we have taken all we can from life	Father
Fear that the children will not take this well	Partner, sister, friend
Worried about weight loss from AYA	Mother
Worrying about physical symptoms	Mothers, father
Worrying what will happen once AYA has nothing to do throughout the day	Father
2.8 Adjusting life to AYA	
Afraid to say things that will upset or burden AYA	Partners, friends, sister
AYA chooses to talk to others over me	Friend, sisters, brother,
Frustration because AYA does not understand simple things	Brother
AYA does not give information on hospital appointments	Friend, father
AYA explains simple things to me due to brain damage and it is frustrating	Brother
AYA gives us little information even though I would like more	Sister, friend
Doubting whether to reach out to AYA	Friend
Difficult finding a balance between caregiving and respecting AYA’s independence	Partner, brother
Having to adjust to when AYA wants to talk about the disease	Friends, mother, partner
I am inpatient but do not want to get frustrated with AYA	Brother
I do not want any focus on me since AYA is the patient	Partners
I feel out of place to ask about prognosis	Partner, sister
I had to balance supporting but also letting go	Mother
Pushing myself away to keep the focus on AYA	Mother, partner
We want to talk to someone separately because we do not want to hurt each other	Partner
We have to keep adjusting to the changing disease trajectory	Partners
Since I have had my baby, AYA is not my priority anymore	Sister
Neglecting my own needs and physical difficulties	Mother
**3.** **Social impact**	
3.1 Relationship to friends	
Afraid to lose my friends if AYA passes	Partner
We spend time with friends before treatment	Friend
Friends help us out if we need it	Friend, partners
Friends cannot relate to our situation	Partner
I am happy I get to share my sadness with friends when AYA passes	Friend, partner
We get to catch up because we give updates on a different platform	Partner
I reach out to friends more often for when AYA passes	Partner
My friends support me by talking or doing fun things	Friends, partners, parents, sister
Not able to see my friends as much as I would like to	Partner
Some friends do not reach out anymore	Parents
Some friends get it and some cannot support me well	Mothers, partners,
There is less time to spend on social contacts	Partner
We have gotten closer to family and friends	Partners, mother
With family and friends, I talk about facts and not feelings	Partner, sister, parents
3.2 Relationship to AYA	
I have to adjust to AYA in the relationship	Partners
AYA mostly talks to me because I have been there for the entire disease	Friend
Network updates each other to decrease the burden on the AYA	Friend
Difficulty when AYA tells me more than our other friends	Friend
Difficulty to balance reaching out and burdening AYA too much	Friend
I brought AYA something to do so we have something to talk about	Brother
AYA does not let me talk about the disease to others	Mother
I know when I can(not) ask AYA about the disease	Father, friends
I try not to only talk about the disease to AYA	Friend, partner, parents
We want to know different information	Partners, mother, friend, sisters,
I would like to share our story, but AYA does not	Mother
We are experiencing a different journey	Partners, mother
We talk a lot about what is happening	Mothers, partners, friends, sisters
It feels like we are missing out on our relationship because of surgery and its recovery	Partner
Because I do not work, I can spend more time with AYA	Mother
I do not know what to say after receiving bad news, so I do not reach out to AYA	Friend
I do not cancel plans with AYA because of the limited time we have left	Partner
I quit hobbies	Father
We isolate from others because I do not want them to infect AYA	Partner, parents
3.3 Positive support	
We get support by asking how we are doing	Mothers, partners
It is nice to talk to others dealing with the same situation	Partners, sister, parents,
I can talk about the disease with my colleagues because they have medical knowledge	Partners, sisters, father
It is easier to talk to people I am not close with	Partner
It Is nice when people reach out to you	Father, partner
3.4 Communication	
Annoying when others do not remember what we have already told them	Partner
We cannot relate to peers since they are already declining	Partner, mother
Our children do not want others to know, and we cannot tell them	Partner, mother
Difficulty communicating to others	Partner, mothers
We had to explain to our children what is going on	Partners, mother
We want to tell our children the truth to give time to process	Partner
It is confronting when others keep asking about the patient	Sister
Others are happier with the positive scan result	Partners
Others avoid talking about the disease	Parents
Others do not tell us everything to avoid burdening us	Partner
Others expect me to distract AYA	Sister
Others think I am strong and doing well because I can talk about it	Partner
Others think they know what is best for our relationship instead of listening to us	Partner
I feel pressured to undergo genetic testing	Partner
Social support decreases as it has been longer since diagnosis	Mothers, partner
Telling others is difficult because of the poor prognosis	Parents
We want to be normal but receive pity	Friend, mother
We cannot relate to others in curative stadium or older patients	Partners, mother
We do not talk to our children about the disease	Partner
We have to set boundaries to others and keep explaining AYA is not recovered	Partner
We have to discuss caregiving for when AYA will decline	Mother
We try and give our children clear information	Partners
My employer did not support me	Mother
Others do not see the impact it has on our life in-between scans	Mother, partner
I have no-one with whom I can share everything	Partner
I feel like I burden others when I talk about the disease	Sister
I cannot talk to others because they do not understand	Mother, partners
I do not want to be involved in support groups because I do not want others to lean on me	Partner
**4.** **Schedule impact**	
4.1 Different tasks to perform	
Accompanying patient to hospital or scans	Sister, brother, parents, friends, partners
Having to do all household tasks by myself	Partners
Having to take care of the children by myself	Partners
Trying to keep stimuli away from AYA	Mother, partner
Preparing for hospital visits and make lists of questions	Partners
Translating medical information	Sisters
Navigating conversations with the doctor	Partners
Distracting AYA or doing fun things together	Sisters, brother, partners, friends, parents
Taking care of AYA’s children	Sister, parents
Taking over when everything gets too much for AYA	Brother
Having to remind AYA to take medication	Partner
Planning everything so AYA can take a rest	Partner
4.2 Changing plans for the future	
Adjusting work schedule to take care of the children	Partners
Changed future because of having to take care of AYAs children after death	Parents
Changing or skip plans to take care of AYA	Mother
Getting married to ease decision making	Partner, sister
No longer being able to do things spontaneously	Partners
Needing a wheelchair accessible house	Partners
Stopping work (temporarily)	Sister, partner
Not starting an education because it cannot be combined with caregiving	Partners
Having to live with AYA after treatment	Friend
Got pregnant quicker so AYA was still able to meet the baby	Sister
Not moving abroad	Partner, sister
Not doing certain activities to be able to provide support if needed	Mothers, father
Study delay because of caregiving	Partner
Can no longer travel	Partners
Cannot move house	Partners
Getting married quicker so AYA can still be present	Sister
Not being able to plan too far ahead	Mothers, partners, sister
Cannot have another child	Partners
Having to speed up the process of having another child	Partners
4.3 Difficulties	
Having to constantly consider everything	Partners
Not having any time to yourself	Partner
Hard to take care of children and AYA at the same time	Partner
Difficulty of being both a partner and caregiver	Partner
Difficulty balancing employment and domestic labor	Partners
Difficult to live life to the fullest, while also dealing with physical symptoms	Partner
**5.** **Financial impact**	
5.1 Worrying about income	
Benefit is not enough to cover one income	Partner
Income does not contribute enough to make ends meet	Partner
Fear I cannot make it financially	Partners
I have to find a job that will give us both enough income	Partners
5.2 Wanting a change	
I want to spend more time with AYA but have to gain income	Partner
I would like to work less hours but cannot do that financially	Partners, mother
We decided to get married to ease financial arrangements	Partner, sister
Glad AYA keeps his salary, so I do not have to work more hours	Partner
5.3 Financial difficulties	
It is complicated to arrange a benefit	Mother, partner
We cannot get a mortgage	Partners
We have to rent a house and cannot buy one, because we cannot get a mortgage	Partner
We cannot do as much because of our reduced income	Partner
We have to sell our house because of the expensive mortgage	Partners

**Table A3:** Quotes derived from the interviews.

Domain	Quote Number	Quote
Biological	1.1	For me, the interest [in sex] has also changed a lot. Because basically, he’s now turned into a very fragile person. […] But it’s just someone undergoing very intense treatments, plus he looks completely different. *(F, 25, partner)*
Psychological	2.1	In the beginning mostly anger of: “why her, why not me?” Our family has dealt with cancer a lot. Both of my parents died from it. So, it was my turn and not hers, in a manner of speaking. That was my mindset at the time. I can’t, I just can’t stand the thought of that. […] No, I still do think it’s unreal. But yeah, like I said before, I’m going, I can’t imagine it yet, and I don’t want to. I very consciously pull away from it. *(F, 64, mother)*
Psychological	2.2	The difficult thing with us is that we have been doing this for five years now, that for five years your life is basically on hold. And of course, because I’m currently in my thirties, at a given moment you have to think about certain things. So, if it [the treatment] had worked, yes, then you feel you can go on with your life and make bigger plans. *(F, 30, partner)*
Psychological	2.3	Because you don’t know what to consider. Look, if they were 100 percent sure of this is how many years she has left. Yeah, then… On the one hand, you wouldn’t want to know either, but at least you know where you stand. And now you don’t know if the time has come, or maybe in ten years. That’s a completely different mindset you’re in regarding how your daily life… Or how you manage and function. And not necessarily on a specific thing, but just, what keeps you occupied all day. It does just keep you preoccupied throughout the day and if you just have a down day, that has a lot of impact on your emotions, so to speak. *(M, 26, partner)*
Psychological	2.4	And I really try to do that too, because it happens sometimes, too, you know, that you just really spiral into this kind of: yeah [swearing] how will it be in ten years? And especially for those kids, I had that at the very beginning, that I think: [swearing] yes. […] And [child] is ten now and was seven then. I say: well, if he doesn’t make those ten years, then you’re talking about eight years. Yeah, then he’ll be 15. I say: I can already see him doing drugs with such a terrible childhood, you know. And how you then… a very vulnerable age. *(F, 36, partner)*
Psychological	2.5	Yes, I will never become a father with [patient]. And we did then, before her diagnosis, we did discuss gosh, that we, she’s 39 now, I’m 38 and we were always like, well… […] Only when I realized that it was quite clear that, yes, that a pregnancy or things like that, yes that that just wasn’t going to happen. *(M, 38, partner)*
Social	3.1	There are some misconceptions in that from time to time, and that is also sometimes inconvenient. That does cause some friction sometimes. And yes, so, it’s not easy either. *(M, 38, partner)*
Social	3.2.	This is so different. One treatment and she’s done. Yes, I can really… I kind of refer to that as the easy kind. Whereas it’s still, don’t get me wrong, it’s still horrible. But it’s not what we’re dealing with. Yes, I would give anything to get that. So you go there, and you’re cured. *(F, 25, partner)*
Social	3.3	In principle, everyone is still engaged, but of course what you will see is that others move on with their lives a bit and sometimes forget what it is like for us. Also, because when they see [patient], they often have the impression: oh, you’re doing great, you look great. *(F, 30, partner)*
Social	3.4	And you realize, yes, I don’t know, I think when you’re sick, you feel lonely quite often. Because also, of course, I don’t know what he’s going through. And my fear and my sadness, that’s different from his fears and his sadness. And so, I think you feel alone quite often too, because… I too will not understand him in that, so to speak, what he is going through. Because, of course, I have my own experience. Or in my own way, say, experiencing his illness. From the other side. *(F, 33, partner)*
Schedule	4.1	We look at it month by month really. So, a year from now, I’ll be done with my PhD and then others will say, “What are you going to do afterwards? And then I think: yes, I’m not even going to think about that now, because I can think about it now, but anything can change. So, then I can really look forward to something that won’t happen. Yeah, why would I do that? So basically, you just change the focus. (*F, 30, partner)*
Schedule	4.2	Yeah gosh, because then when I look at it, a day has 24 h and I work between 8 and 10 of them. The remainder of the time is spent on family life, on things that must be done around the house, and that does mean that I have very little time to myself. That could be as little as 2 h. And then I’m fortunate that I don’t need a lot of sleep, I sleep 5 to 6 h a day. If I didn’t, I would be facing a problem. *(M, 38, partner)*
Schedule	4.3	Because with getting married came… We had figured: let’s do that because that will be nice. Also, because he really prefers that if he cannot make any more decisions, he wants me to do it. So that is a conversation we have had. And of course, with bank affairs et cetera. Thus, that all his bank affairs have been shared and I am on them and have access to them. *(F, 30, partner)*
Schedule	4.4	We, my partner and I, do it with love and enjoy it of course. Because I mean, he also is our everything, as yes, that how it works with grandchildren. But that does mean that you in your, yeah well, we are both still relatively, we were young parents, so we are young grandpa and grandma. […] But that does implicate for you that, if it would be the case, that you, to be, how do you say it, enjoy retirement is of course, will be different. Will be different at that time. *(M, 55, father)*
Financial	5.1	That I had a hard time finding a job and that I did not feel well emotionally. So that caused a lot of stress. And still, I must earn enough money and need enough work to be able to do so. *(F, 24, partner)*
Financial	5.2	Of course, I only want to do fun things with [patient]. You know, sometimes it is difficult to find a balance as that does not seem possible. And when your employer also gives you a hard time, then all your energy and attention is focused on the wrong things. Then there is no room for that, and I find that very difficult. And you know it is for a good cause, because this is what you want. But because of the struggle at work, I find it most difficult I do not have quality time with [patient], which I would like to have. *(F, 45, partner)*
Financial	5.3	Yes, I have written the municipality. But as I work full-time and have too much income, according to the norm, we could in a… Look, we have … we have a nice house. We made that decision ourselves. But also that, we still have a rental house because a mortgage is very difficult to get with someone who will never be 100 years old. And who does not have a permanent employment. Yes, I worry about that sometimes, where I think: [swearing], why would we… We have been paying this amount of money for years. Why are we not able to buy a house? *(F, 36 partner)*
